# Roles of GSK3β in Odor Habituation and Spontaneous Neural Activity of the Mouse Olfactory Bulb

**DOI:** 10.1371/journal.pone.0063598

**Published:** 2013-05-03

**Authors:** Zhixiang Xu, Li Wang, Guo Chen, Xiaoping Rao, Fuqiang Xu

**Affiliations:** 1 State Key Laboratory of Magnetic Resonance and Atomic and Molecular Physics, Wuhan Institute of Physics and Mathematics, the Chinese Academy of Sciences, Wuhan, China; 2 University of Chinese Academy of Sciences, Beijing, China; 3 College of Life Sciences, Wuhan University, Wuhan, China; 4 Wuhan National Laboratory for Optoelectronics, Wuhan, China; Université Lyon, France

## Abstract

Glycogen synthase kinase 3β (GSK3β), a multifaceted kinase, is abundantly expressed in the brain, including the olfactory bulb (OB). In resting cells, GSK3β is constitutively active, and its over-activation is presumably involved in numerous brain diseases, such as Alzheimer’s disease. However, the functions of the constitutively active GSK3β in the adult brain under physiological conditions are not well understood. Here, we studied the possible functions of GSK3β activity in the OB. Odor stimulation, or blockade of peripheral olfactory inputs caused by either transgenic knock-out or ZnSO4 irrigation to the olfactory epithelium, all affected the expression level of GSK3β in the OB. When GSK3β activity was reduced by a selective inhibitor, the spontaneous oscillatory activity was significantly decreased in the granule cell layer of the OB. Furthermore, local inhibition of GSK3β activity in the OB significantly impaired the odor habituation ability. These results suggest that GSK3β plays important roles in both spontaneous neural activity and odor information processing in the OB, deepening our understanding of the potential functions of the constitutively active GSK3β in the brain under physiological conditions.

## Introduction

Glycogen synthase kinase 3β (GSK3β), a conserved serine/threonine protein kinase, that is abundantly expressed in the mammalian brain [1], [2]. The activity of this kinase is primarily regulated by phosphorylation. Phosphorylation at Ser9 (S9-GSK3β) and Tyr216 (Y216-GSK3β) inhibits and activates the kinase activity, respectively. In resting cells, Y216-GSK3β is constitutively phosphorylated [3], and the activity of GSK3β is primarily regulated by inhibition. Studies have revealed that GSK3β activity might play roles in neural activity. For example, GSK3β is essential for activity-dependent bulk endocytosis of synaptic vesicles during elevated neural activity through re-phosphorylation of Dynamin I [4]. Consistent with this observation, it has been reported that GSK3β is involved in the regulation of the two major forms of synaptic plasticity, long-term potentiation (LTP) and long-term depression (LTD) [5], [6], [7]. Accordingly, dysregulation of GSK3β activity in the brain is involved in many neurological diseases and psychiatric disorders, such as Alzheimer’s disease, schizophrenia and bipolar disorder [2], [8]. However, the functions of the constitutively active GSK3β in the adult central nervous system (CNS) under physiological conditions are not well studied.

The olfactory system is crucial for animals. Olfaction is involved in a wide range of behaviors, including emotional modulations, mate selection, sexual and parental behaviors, aggressive behavior, etc [9], [10], [11]. It is initiated when odorants bind to their receptors in the olfactory sensory neurons (OSNs) which triggering a transduction cascade that results in the release of neurotransmitters, such as glutamate, into the glomeruli [9]. Odorant information is further processed in the olfactory bulb (OB) and sent to the primary olfactory cortices by mitral/tufted cells. Previous studies have reported that the synthesis and release of neurotransmitters in the OB are activity-dependent [9], [12], [13], [14] and olfactory dysfunction is associated with many neurological diseases, such as Alzheimer’s disease, Parkinson’s disease and schizophrenia [15], [16], [17], [18], although the roles of olfaction in these neurological diseases are not yet clear.

GSK3β is abundantly expressed in the OB, including the periglomerular cells, mitral cells and granule cells (Fig. S1). It regulates the axonal stability of the OSNs [19], and its activated form (Y216-GSK3β) was abundantly detected in the adult OB (Fig. S2). Base on the functions of GSK3β for synaptic vesicles retrieval in neuronal cultures [4], the regulation of LTP and LTD by GSK3β [5], [6], [7], the expression pattern of GSK3β and constitutive phosphorylation of Y216-GSK3β in the olfactory bulb (Figs. S1 and S2), we hypothesize that GSK3β is activity-dependent and plays important roles in olfactory functions under physiological conditions.

To test our hypotheses, we manipulated the peripheral inputs to the OB through odor deprivation or odor exposure in our studies and found that the activity of GSK3β indeed was neural activity-dependent. Furthermore, we altered the activity of GSK3β by using its specific inhibitor and found that the spontaneous neural activity in the OB was significantly decreased and that the odor cross-habituation behavior was significantly impaired. These results demonstrated that this kinase is involved in more general neural processes, providing evidences why its dys-regulation could lead to a variety of brain diseases.

## Results

### Expression and activity of GSK3β are dependent on odor-evoked neural activity

**Figure 1:**
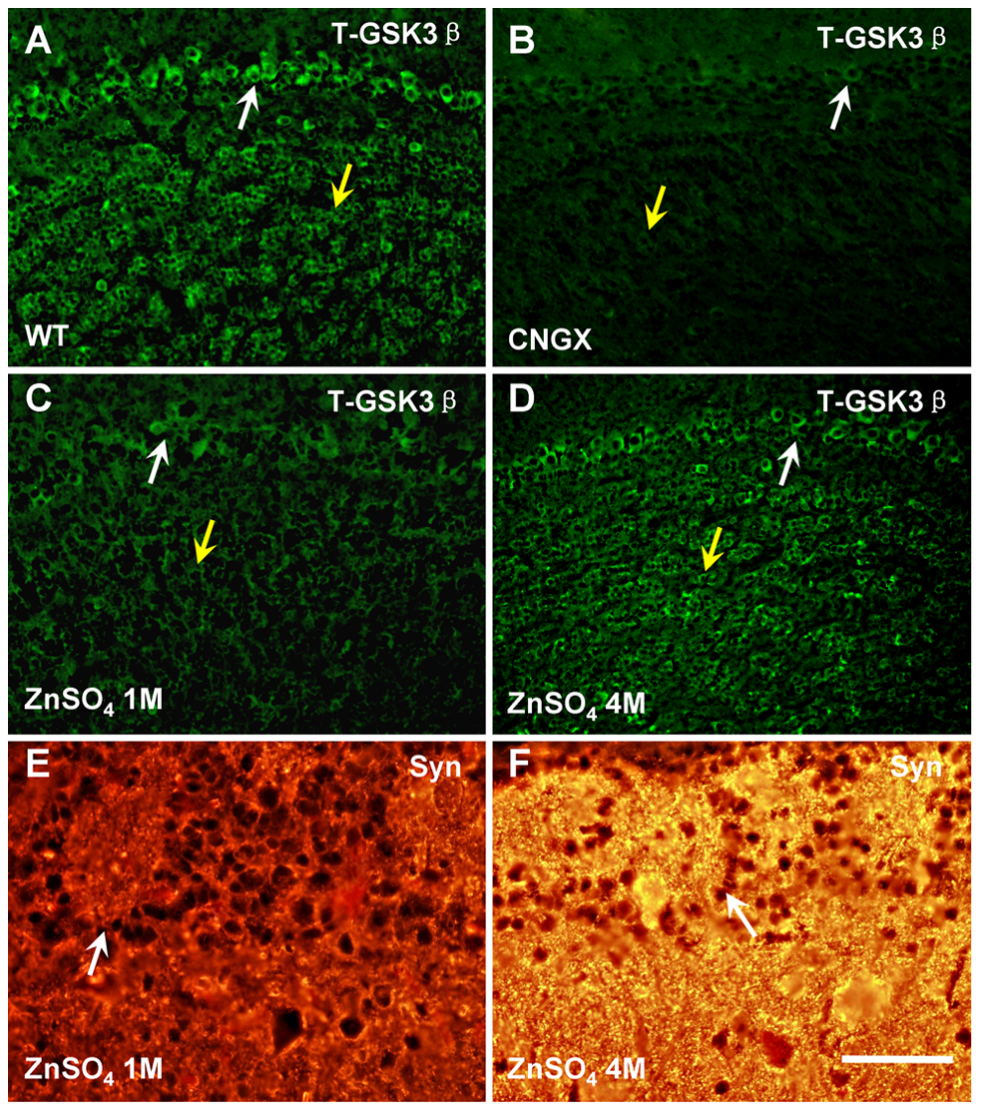
Olfactory deficit reduced the expression level of GSK3β in the OB. A: T-GSK3β was widely expressed in the mouse OB, including the mitral cells and granule cells. B: The T-GSK3β signal was significantly decreased in the OBs of CNGX mice. C: The T-GSK3β signal was decreased one month after ZnSO_4_ irrigation. D: The T-GSK3β expression level in OB was partially recovered four months after ZnSO_4_ irrigation. (A–D: white arrow, mitral cell layer; yellow arrow, granular cell layer). E & F: Compared to the signal at one month following ZnSO_4_ irrigation, Syn in the OB was stronger at four months post-treatment (E–F: white arrow, glomerulus). Syn: synaptophysin; Scale bar, A–D, 100 µm; E–F, 50 µm.

**Figure 2:**
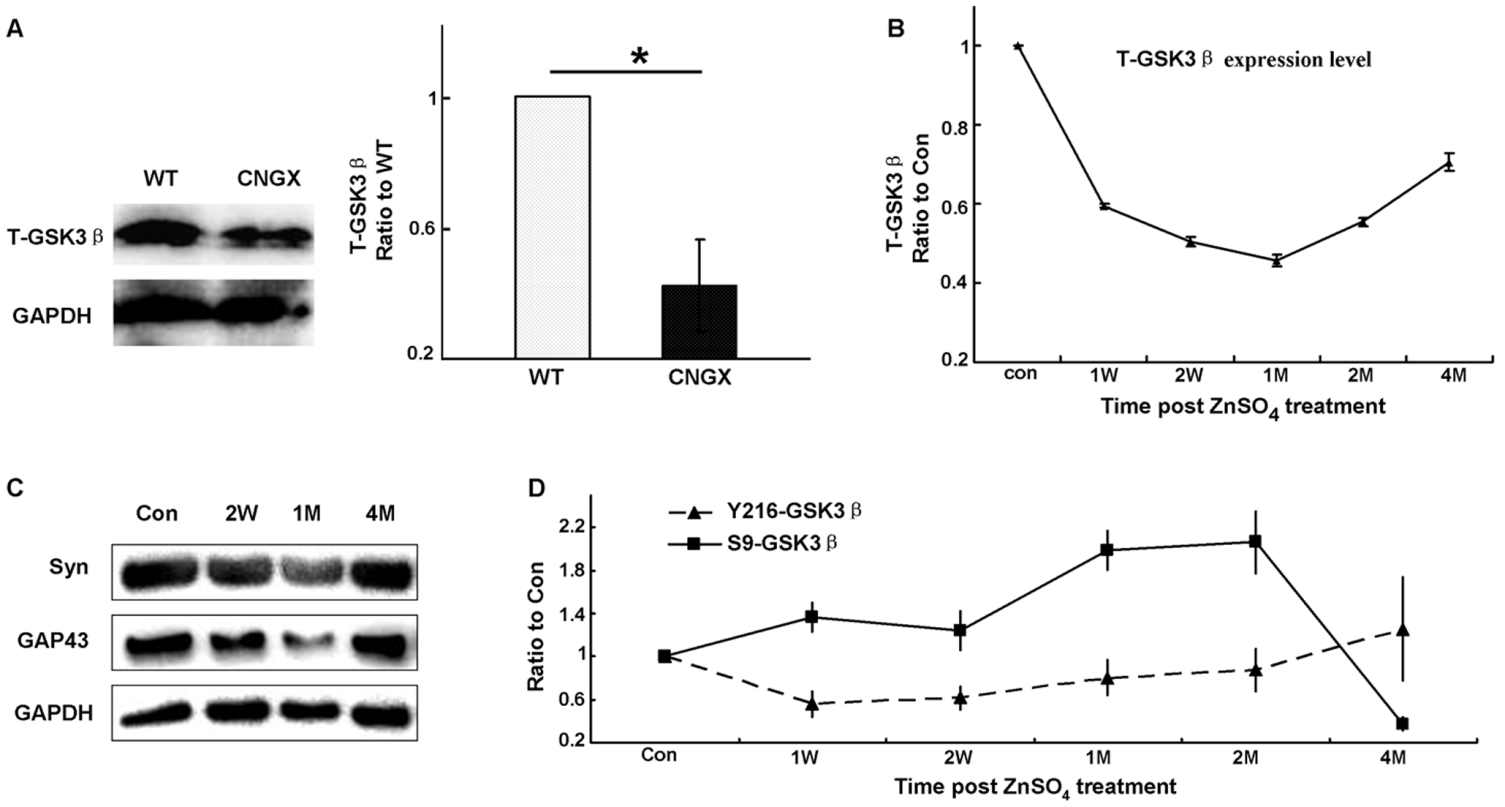
Western blot analysis of GSK3β expression level and activity in the OBs of olfactory-defective mice. A: Western blot revealed that the T-GSK3β expression level was significantly decreased in OBs from CNGX mice (n = 6, P<0.05). B: The T-GSK3β expression level changed dynamically during the ZnSO_4_ irrigation and recovery stages (ratio to control, n = 5–6). C: Western blot revealed that Syn and GAP43 were reduced in the OB at 2 weeks and 1 month post-irrigation, and then increased 4 months post-ZnSO_4_ irrigation. D: The expression patterns of Y216-GSK3β (ratio to control, n = 5–6 mice) and S9-GSK3β (ratio to control, n = 5–6) in the OB following ZnSO_4_ irrigation. Syn, synaptophysin; GAP43, growth associated protein 43. * P<0.05. The data shown are the average ± SEM.

If constitutively active GSK3β plays important roles in the neural activity, its expression in OB should be affected accordingly by the conditions of olfactory sensory inputs. To test this assumption, transgenic mice (Cyclic nucleotide gated channel 2 knockout, CNGX) were used [20], [21]. The ion channel is necessary for OSNs to generate odor induced action potentials, rendering CNGX mice essentially anosmic. Using immunohistochemistry (IHC), we found that the signal of total-GSK3β (T-GSK3β) in the OB of CNGX mice was remarkably decreased in the mitral cell and granule cell layers (Fig. 1A–B). To provide quantitative information and confirm the IHC results, Western blotting analysis was performed. Compared with the WT OBs, there was indeed a significant reduction of T-GSK3β signal in the CNGX OBs (Fig. 2A; n = 6 in each group, P<0.05).

To preclude the effects caused by genetic alteration in CNGX animals, we next analyzed the expression level of GSK3β in another anosmic model. Intranasal irrigation with ZnSO_4_ solution causes acute olfactory epithelium destruction, leading to a recoverable anosmia because of the continuous generation of OSNs from basal cells [22], [23]. In this model, we found that the expression level of T-GSK3β was first decreased one month post-ZnSO_4_ irrigation and then increased after four months (Fig. 1C–D). Consistent with the IHC studies, Western blot analysis showed the same expression tendencies of T-GSK3β during the ZnSO_4_ irrigation and recovery course (Fig. 2B). To confirm that this dynamic change in T-GSK3β was due to the loss/regaining of olfactory sensory input, we analyzed the expression levels of synaptophysin (Syn) and growth associated protein 43 (GAP43) during the process. The two proteins have been previous used as markers for recovery after zinc sulfate irrigation of OE [24]. We found that the punctuate signal of Syn was stronger in the OB four months post-irrigation compared to the one month post-irrigation (Fig. 1E–F). Western blot analysis also demonstrated Syn and GAP43 were reduced in the OB at 2 weeks and 1 month post-irrigation, and then nearly recovered to the pre-treatment level (Fig. 2C, Fig. S3). Thus, these results suggest that the expression level of GSK3β is dependent on peripheral inputs and the neural activity in the network.

The activity of GSK3β is primarily regulated by phosphorylation [2], [25], [26]. To reveal not only the total protein expression level, but also the phosphorylation status of GSK3β were affected during the irrigation/recovery procedures; we used specific anti-S9-GSK3β (the inactive form) and anti-Y216-GSK3β (the active form) antibodies to determine the amounts of corresponding forms. By Western blot analysis, we found that Y216-GSK3β decreased to its lowest level one week post-ZnSO_4_ irrigation and then increased gradually during the recovery process (Fig. 2D). Unlike T-GSK3β and Y216-GSK3β, S9-GSK3β showed the opposite tendency during the same time course (Fig. 2D). Thus, both the expression level and activity of GSK3β were dependent on odor-evoked neural activity.

### Odor exposures increase the activity of GSK3β without altering the expression level

**Figure 3:**
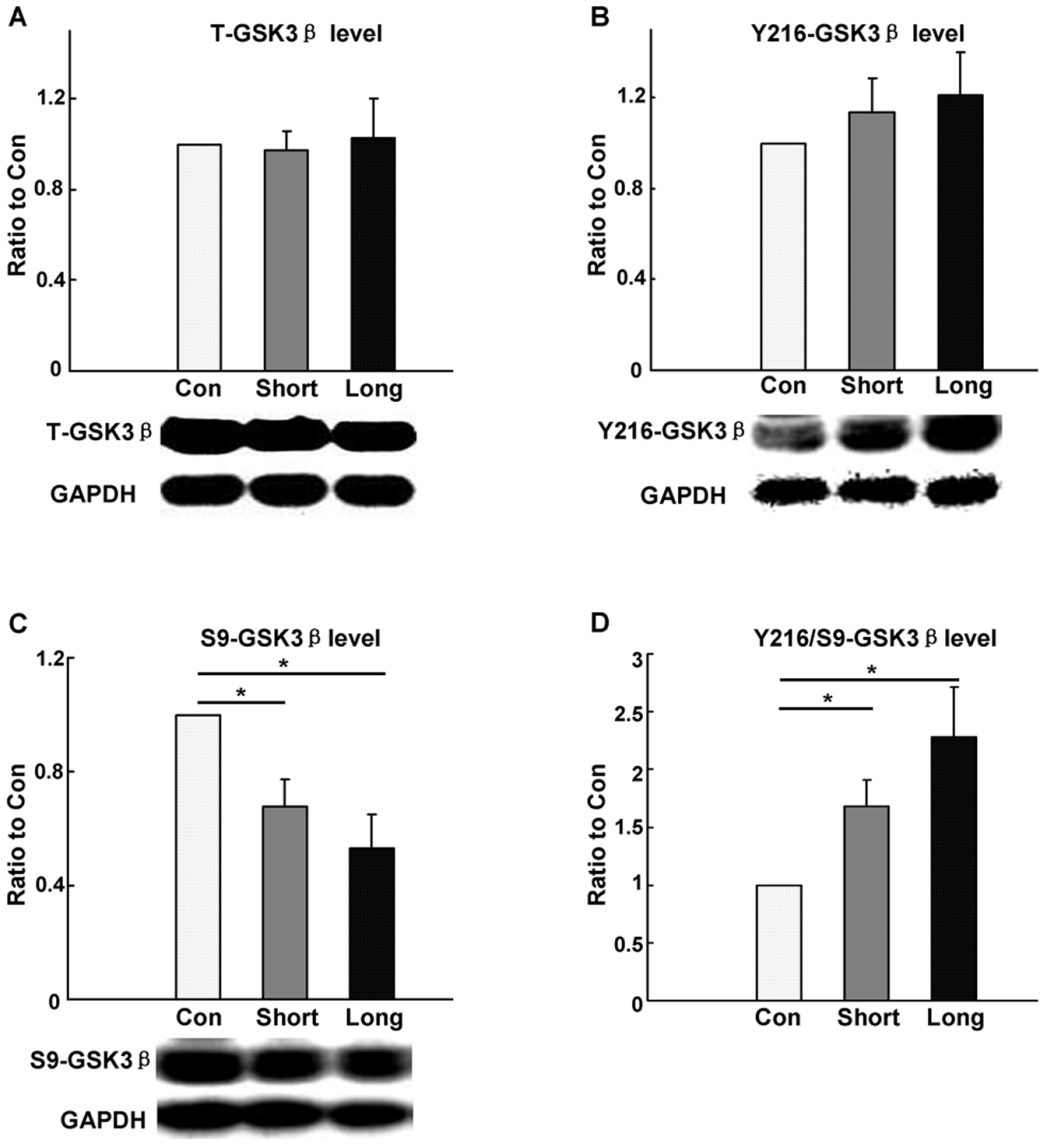
Western blotting analysis of odor exposure on GSK3β activity in the OB. A: T-GSK3β expression level was not altered in the three treated groups (n = 8, P_(C to S)_ >0.05; P_(C to L)_ >0.05). B: Y216-GSK3β showed an increasing tendency in short-term and long-term enriched odor exposure OBs (n = 8, P_(C to S)_  = 0.131; P_(C to L)_  = 0.214). C: S9-GSK3β presented a decreasing tendency in the OBs from mice subjected to short-term and long-term enriched odor exposure (n = 8, P_(C to S)_ <0.05; P_(C to L)_ <0.05). D: Phosphorylation status expressed as the ratio of Y216-GSK3β to S9-GSK3β (Y216-GSK3β/S9-GSK3β) significantly up-regulated after short- and long- term odor exposures (P_(C to S)_ <0.05; P_(C to L)_ <0.05). C: control; S: short-term; L: long-term. * P<0.05. The group data shown are the average ± SEM.

Because olfactory deficits down-regulated the expression level and activity of GSK3β in the OB, we further tested whether odor exposure could generate the opposite effects on GSK3β (i.e., increasing expression level and activity). T-GSK3β in the OBs were not changed in any of the conditions, including the control, short- and long-term odor exposures (Fig. 3A, 8 mice in each group, P_(C to S)_ >0.05; P_(C to L)_ >0.05). Then, we tested whether the GSK3β activity is changed as a result of odor exposure. Y216-GSK3β tended to increase after short- and long-term odor exposures, although differences between the groups were not significant (Fig. 3B, 8 mice in each group, P_(C to S)_  = 0.131; P_(C to L)_  = 0.214). Conversely, S9-GSK3β showed the trend to decrease after short- and long-term exposures (Fig. 3C, significant for short- and long-term exposures, 8 mice in each group, P_(C to S)_ <0.05; P_(C to L)_ <0.05). Moreover, the phosphorylation status evaluated by the ratio of Y216-GSK3β to S9-GSK3β (Y216-GSK3β/S9-GSK3β) was significantly up-regulated after short- and long- term odor exposures (Fig. 3D, P_(C to S)_ <0.05; P_(C to L)_ <0.05). These findings demonstrate that GSK3β activity is increased a result of the elevated neural activity, as we have expected.

### Inhibition of GSK3β decreases the spontaneous neural activity in the OB

**Figure 4:**
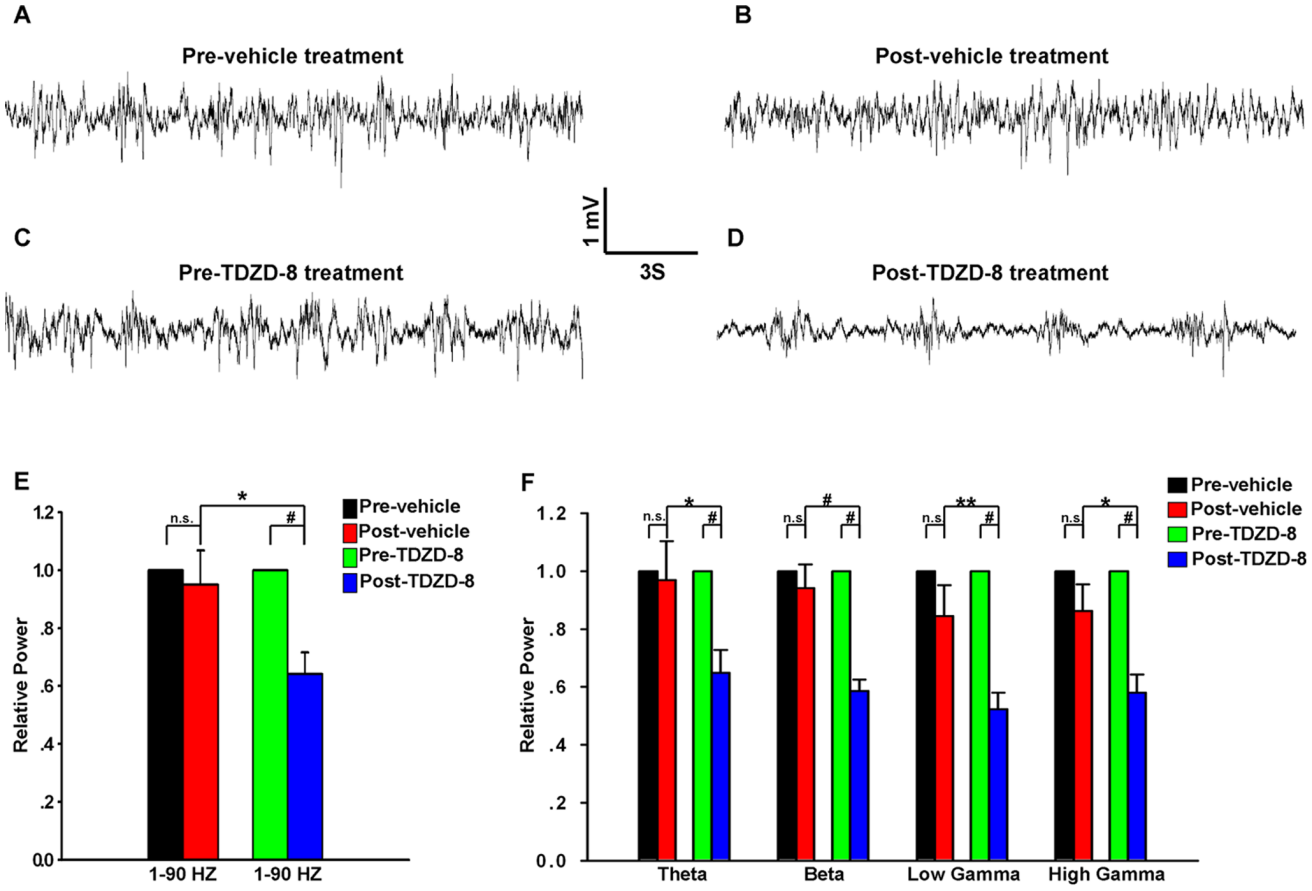
Electrophysiological recordings of the spontaneous oscillatory activity in the granular cell layer of the OB. A–D: Raw data displaying representative examples of spontaneous LFP recordings. A & B show the raw spontaneous LFP signals in pre- and post-vehicle treated OB from the same mouse, respectively. C & D presents the raw LFP signal from a pre- and post-TDZD-8-treated mouse, respectively. E shows the changes in relative power (1–90 Hz) in the vehicle- and TDZD-8- treated groups. There is significant difference between the OBs of mice before and after TDZD-8 treatment (P<0.001), but no significant difference between the pre- and post-vehicle-treated mice (P = 0.685); significant difference between post-vehicle and TDZD-8 treatment (P = 0.028). F: The relative power of the four frequency bands did not show significant differences between pre- and post-vehicle treatment (all, P>0.05). The pre- and post-TDZD-8-treated mice had significant differences in the four frequency bands (all, P<0.001); significant difference between post vehicle and TDZD-8 treatment (Theta, P = 0.037; Beta, P<0.001; low Gamma, P = 0.008; high Gamma, P = 0.014). The data obtained from the GCL in the vehicle-treated (10 mice) and TDZD-8 (16 mice). Not significant (n.s.). * P<0.05; ** P<0.01; # P<0.001. The group data shown are the average ± SEM.

GSK3β is constitutively active in adult mouse OB (Fig. S2), and the above results have demonstrated that neural activity and GSK3β activity are correlated. Based on these facts, we hypothesized that the spontaneous neural activity in the OB would be reduced, if the activity of GSK3β was suppressed. We used local field potential (LFP) oscillation as a measurement of spontaneous activity of neuron population. To limit the effects of feedback and modulatory inputs to the OB from the higher brain regions, our electrophysiological recordings were performed in anesthetized mice. We found that the power of spontaneous baseline activity (1–90 Hz) in the OB was significantly decreased (Fig. 4C, D and E, P<0.001, 16 mice), after the animals were treated with TDZD-8, a specific inhibitor of GSK3β activity [27], while the spontaneous neural activity in the vehicle-treated mice was not altered (Fig. 4A, B and E, P = 0.685, 10 mice). In addition, there was significant difference between post- vehicle and TDZD-8 treatment mice (Fig. 4E, P = 0.028). We further assessed the power in different frequency components of the LFP separately. Fig. 4F showed that all the four frequency bands were significantly decreased after TDZD-8 treatment (Theta, 1–12 Hz; Beta, 12–35 Hz; low Gamma, 35–60 Hz; high Gamma, 60–90 Hz; all P<0.001); in addition, significant differences were observed in the post-vehicle and post-TDZD-8 treatment mice (Fig. 4F, Fig. S4; all, P<0.05). However, there were no significant differences in these four frequency bands of the vehicle-treated mice (Fig. 4F; P>0.05 for all). Thus, selective inhibition of GSK3β activity indeed can decrease the spontaneous neural activity in the OB.

### Selective inhibition of GSK3β in the OB impairs odor cross-habituation

**Figure 5:**
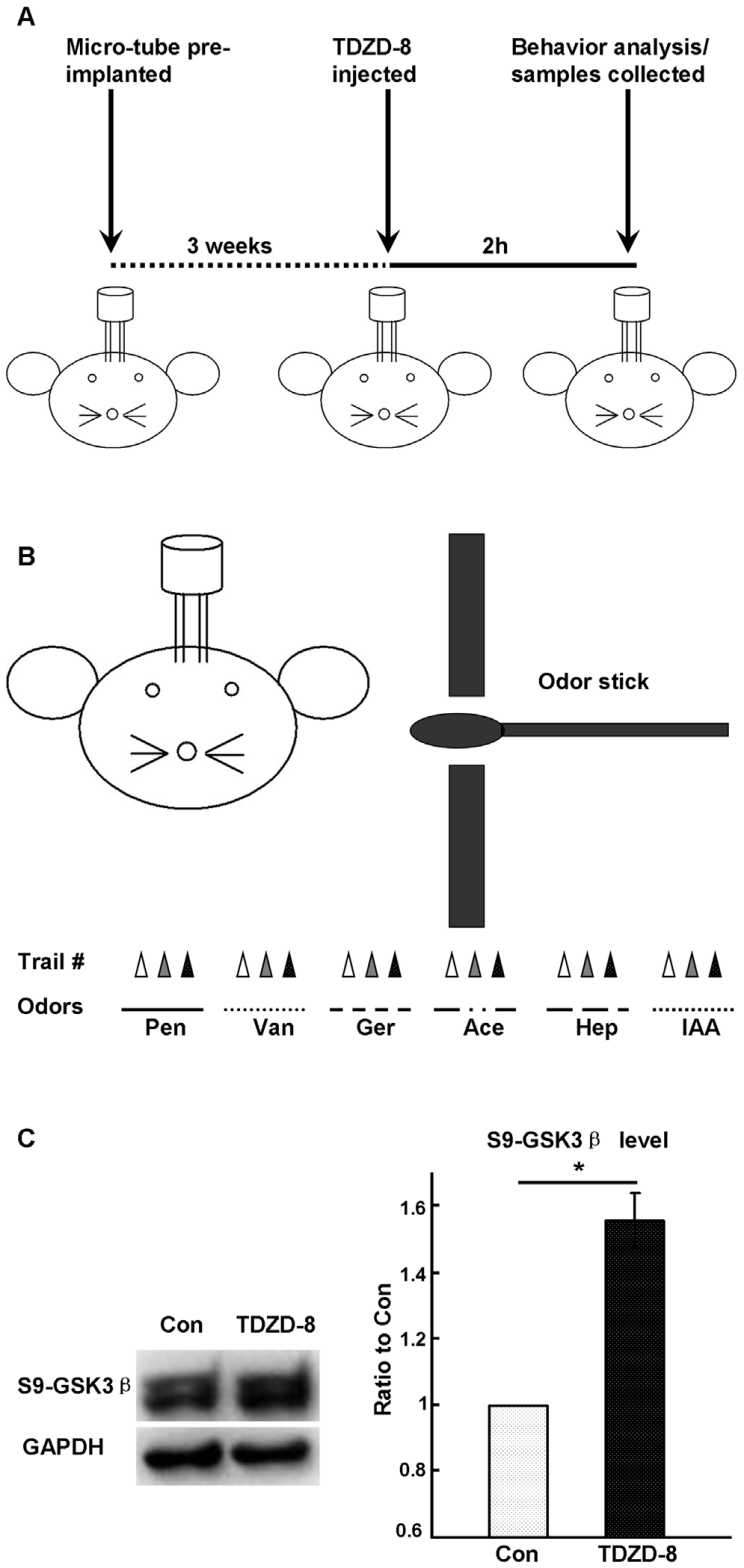
Scheme of the procedure for the animal behavior testing. The mice were bilaterally implanted with stainless steel cannulas into OB and allowed to recover for 3 weeks. Two hours before the behavioral testing, TDZD-8 or vehicle was delivered into the OB through the cannula (A), and the odor cross-habituation behavior analysis was performed following a standard protocol (B). C: S9-GSK3β was significantly up-regulated in the OBs of TDZD-8-treated mice (n = 8, P<0.05). (n-pentanol, Pen; vanillin, Van; geranialdehyde, Ger; acetophenone, Ace; Heptanone, Hep; Isoamyl Acetate, IAA). * P<0.05. The data shown are the average ± SEM.

**Figure 6:**
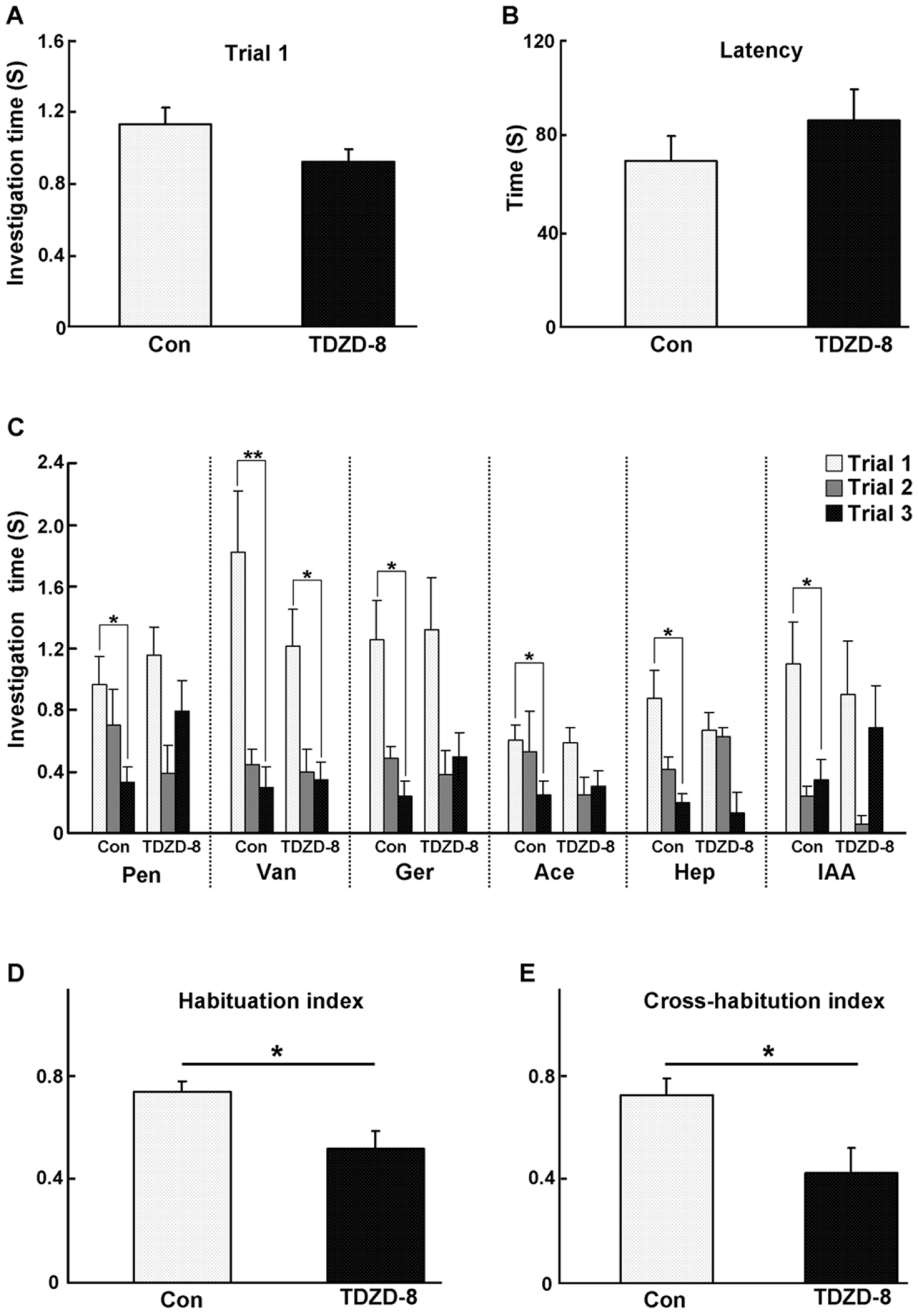
Selective inhibition of GSK3β activity in the OB impairs odor cross-habituation. A: The investigation time of trial one in each block (each odor) did not show a statistically significant difference between the TDZD-8 and vehicle-treated group (P>0.05). B: The latencies of each block in the TDZD-8- and vehicle-treated animals were not significantly different (P>0.05). C: The investigation time of each block and each trial. The TDZD-8 treated group displayed habituation only to vanillin, while the vehicle-treated group habituated to all six of the tested odors. D & E: Compared to the vehicle-treated animals, the habituation index (P<0.05) and odor cross-habituation index (P<0.05) in the TDZD-8-treated animals decreased significantly. The data were obtained from 16 animals in the vehicle group and 12 animals in the TDZD-8 group. * P<0.05, ** P<0.01. The group data shown are the average ± SEM.

Since GSK3β activity was dependent on neural activity and its inhibition reduced the spontaneous neural activity, the kinase activity might play roles in olfactory information processing. Thus, we tested the potential functions of GSK3β in olfaction using behavioral analysis. Mice were bilaterally implanted with stainless cannulas into the OBs and allowed to recover for 3 weeks. After that we locally inhibited the activity of GSK3β with TDZD-8 and assessed the olfactory performance by odor cross-habituation test (Fig. 5). The investigation time (Fig. 6A, P>0.05) and latency (Fig. 6B, P>0.05) of trial one in each block for each odor did not show statistically significant differences between the vehicle- and TDZD-8-treated groups, suggesting that these mice can detect and discriminate odors normally. However, when analyzing the habituation process in each block, we found that the TDZD-8-treated mice habituated normally to only one odor, while the vehicle-treated mice habituated to all six of the tested odors (Fig. 6C). Moreover, the habituation index (Fig. 6D, P<0.05) and cross-habituation index (Fig. 6E, P<0.05) were significantly different between the vehicle- and the TDZD-8-treated groups. These findings demonstrated that mice could detect and discriminate odors after the inhibition of GSK3β; however, their discrimination and habituation abilities were significantly impaired. Thus, our findings suggest that GSK3β is required for normal olfactory functions.

## Discussion

### Roles of GSK3β in neural activity

GSK3β is an important component of many intracellular signaling pathways, thus its activity must be precisely regulated [2]. In the adult OB, GSK3β was constitutively active (Fig. S2), suggesting its involvement in fundamental cellular processes. In the present study, using odor deprivation models (CNGX and ZnSO_4_ irrigation, Fig. 1A–D and Fig. 2) and odor exposure (Fig. 3), we have demonstrated that the expression level and activity (phosphorylation status) of GSK3β were dependent on odor-evoked neural activity in the OB. These results suggest that the activity of GSK3β is correlated to odor-evoked neural activity.

Then using electrophysiological recordings, we found that the spontaneous oscillatory activity was decreased in the granular cell layer after the inhibition of GSK3β by TDZD-8 (Fig. 4, Fig. S4), confirming the correlation between GSK3β and neural activity. These findings provide evidences about the constitutively active GSK3β in the OB may play important roles in spontaneous neural activity.

The correlation between neural activity and GSK3β may relate to the potential roles of GSK3β in neurotransmission. Recycling process for synaptic vesicles is essential to maintain neurotransmission in the brain. Activity-dependent bulk endocytosis is the dominant mechanism for synaptic vesicle recycling during/after elevated neural activity [28], [29], [30]. Studies have shown that the activity of GSK3β is essential for activity-dependent bulk endocytosis through re-phosphorylation of dynamin I [4]. Thus, the explanation for the dependence of GSK3β activity on odor-evoked neural activity might be as follows: in the short-term and long-term odor exposures, OB neurons respond to odor with elevated neural activity and rapid synaptic vesicle recycling is required to maintain efficient synaptic transmission. Thus, the activity of GSK3β would be increased. In anosmic animals, for example, odor deprivation as a result of CNGX, ZnSO_4_ irrigation or naris closure causes a substantial reduction in the release of neurotransmitters and the cellular expression of corresponding proteins [9], [12], [13], [14], including GSK3β (Fig. 1 and Fig. 2). Moreover, our proteomics studies of the CNGX OB for phosphorylated proteins have revealed that the expression of many proteins involved in synaptic vesicle recycling were significantly changed compared with the wild type OB (manuscript in preparation). Nonetheless, further studies are necessary to clarify the detailed mechanism of GSK3β in regulating neural activity in the OB and the olfactory system.

### Roles of constitutive activity of GSK3β in odor habituation

When the GSK3β activity was suppressed with a selective inhibitor, the animals’ capability of odor habituation and cross-habituation were significantly impaired (Fig. 6). In this study, habituation-dishabituation, not sniffing time, was used as indicator of olfactory function; therefore our results suggested the impairment of discrimination and habituation abilities in TDZD-8-treated mice. Habituation is a fundamental indicator of the implicit short-term memory, which underlies the basic olfactory discrimination capacities [31], [32], thus our data also suggested that short-term memory was impaired after inhibition of GSK3β.

A genetic screen of impaired habituation behavior in *Drosophila* has found a mutation in *Shaggy*, which encodes a homolog of GSK3β [33]. In the present study, we showed that inhibition of GSK3β impaired the normal habituation ability and short-term memory without altering odor detection. These findings might be due to the roles that GSK3β plays in synaptic plasticity and neurotransmission [4]. A number of reports have laid the foundation for our understanding of a causal link between habituation and synaptic depression [34]. GSK3β activity played an essential role in the induction of LTD [6]. Inhibition of GSK3β with CT99021 relieves short-term synaptic depression, which is due to the contribution of GSK3β in activity dependent bulk endocytosis during neurotransmission [4]. In the olfactory system, studies have reported that neuromodulators (noradrenaline, acetylcholine, etc.) serve important functions in odor habituation and short-term memory; blockade of the neurotransmission in the OB impair the short-term memory without altering odor detection [35], [36], [37], [38]. Thus, our observations of impairment in odor cross-habituation and short-term memory might be related to the affected synaptic neurotransmission procedure through the inhibition of GSK3β activity.

The finding that GSK3β is involved in the habituation process is interesting, considering that many neurological diseases, such as Schizophrenia, have been described to show defects of habituation in a number of paradigms [39]. In addition, many studies have demonstrated that there is a strong implication of structural and functional olfactory deficits in Schizophrenia [17], [40], [41]. Moreover, the protein, mRNA and activity of GSK3β are reduced in the postmortem brains of Schizophrenia patients, suggesting the involvement of GSK3β activity in the etiology of schizophrenia [42], [43]. Thus, the roles of GSK3β activity in habituation are of importance for further study to illustrate possible mechanisms of this kinase in brain diseases.

The sniffing time of the mice in trial one and the latency of each block did not change after the kinase activity was inhibited in the OB (Fig. 6A–B). These results also suggested that the movement and motivation of the TDZD-8-treated animals might not be altered. This is somewhat a surprise, given the important roles of GSK3β in general neural activities. However, this observation might be due to the drug delivery. The specific inhibitor is administrated locally to the OB (Fig. 5A), therefore, only the olfactory functions are affected, while the other brain functions like motivation and motor functions, which are largely dependent on the other brain regions, are maintained.

In summary, our data showed a dependence of GSK3β activity on neural activity, and the constitutive activity of GSK3β was important for spontaneous neural activity and odor habituation in the olfactory system. Our findings provided experimental evidence for the potential roles of constitutively active GSK3β in the brain under physiological conditions. Additional studies are necessary to reveal the exact roles and mechanisms of GSK3β in neural activity, which may help us to understand the potential etiology of some neurological disorders in which GSK3β activity is involved.

## Materials and Methods

### Animals

C57Bl/6 mice were purchased from Wuhan university animal experiment center, Wuhan, China. Cyclic nucleotide gated channel 2 knockout (CNGX) mice were gifts from Dr. Minmin Luo’s lab at the National Institute of Biological Sciences, and genotyped through a standard protocol using tail DNA PCR analysis. All animals were maintained on a 12h/12h light-dark cycle, with food and water available *ad libitum*.

### Ethics Statement

The animal experiments were carried out in strict accordance with the protocols approved by the Animal Ethics Committee at the Wuhan Institute of Physics and Mathematics, Chinese Academy of Sciences (SYXK(E)2009-0051, No. 00006626). All efforts were made to minimize animal suffering.

### Antibodies

The primary antibodies included rabbit anti-GSK3β (Santa Cruz; sc-9166; IHC, 1∶150; WB, 1∶500); rabbit anti-pSer9-GSK3β (Cell Signaling Technology; #9323; WB, 1∶1000); rabbit anti-pTyr216-GSK3β (Santa Cruz; sc-135653; WB, 1∶1000); rabbit anti-growth associated protein 43 (GAP43, Abcam; EP890Y; WB, 1∶10000); mouse anti-synaptophysin (Abcam; ab8049; IHC, 1∶100; WB, 1∶400) and chicken anti-GAPDH (Millipore; AB2302; WB, 1∶6000). The secondary antibodies were FITC-conjugated goat anti-rabbit (KPL, 172-1506, 1∶200); CY3-conjugated goat anti-mouse (Jackson ImmunoResearch, 115-165-003, 1∶500); HRP-conjugated goat anti-rabbit (KPL, 074-1506, 1∶5000); HRP-conjugated goat anti-mouse (KPL, 074-1806, 1∶5000) and HRP-conjugated goat anti-chicken (KPL, 14-24-06, 1∶5000).

### Zinc sulfate irrigation

Intranasal irrigation of ZnSO_4_ was performed on adult mice as described previously [22], [44]. Briefly, two-month-old mice were weighed and anesthetized with intraperitoneal injection of 5% chloral hydrate. A PE50 tube was inserted into the nasal cavity, and the ZnSO_4_ solution was slowly administered with a Quintessential Stereotaxic Injector (QSI, cat NO. 53311, Stoelting Company). The mice were bilaterally subjected to intranasal irrigation with 50 µl of 0.17 M ZnSO_4_/normal saline. Immediately after ZnSO_4_ irrigation, the mice were held with their head down for 30 s to minimize the spread of the solution to the oral cavity. The animals were sacrificed after 1 week, 2 weeks and 1, 2, 4 months for immunohistochemistry or Western blot studies.

### Immunohistochemistry

The mice were anesthetized with intraperitoneal urethane (1.4 g/kg) and then transcardially perfused with PBS (150 ml) and 4% paraformaldehyde (PFA) (50 ml). The OBs were post-fixed in PFA overnight and further cryoprotected with 30% sucrose solution, then sectioned at 20 µm with a freezing microtome (LEICA CM1850, Germany) and attached to glass slides coated with dichromate gelatin. The slides were stored at –80°C. For immunohistochemistry, slides were pre-treated with 0.3% Triton-X in PBS for one hour at room temperature followed by rinsing in PBS for 5 minutes. Then, the sections were blocked in 10% normal goat serum in PBS for 1 hour at room temperature and incubated in diluted primary antibody overnight at 4°C. After washing with PBS (3×5 minutes), the slices were incubated with secondary antibodies for 90 minutes at 37°C and washed with PBS (3×5 minutes). The slides were dehydrated in a series of 80%, 95%, 100%, 100% alcohol solutions, dried and mounted in glycerin mounting medium containing DAPI (1 µg/ml) and DABCO (2%).

### Microscopy

Images were acquired using an Olympus BX51 microscope and analyzed using IMAGE-PRO PLUS (Media Cybernetics) and PHOTOSHOP (Adobe) for balancing the brightness, contrast and overlapping.

### Western blot

One microliter of TDZD-8 (10 mM dissolved in normal saline containing 50% DMSO; Sigma; T8325) or vehicle was bilaterally delivered to the OB through a pre-implanted cannula with a 33-gauge Hamilton syringe. Two hours later, the mice were anesthetized and sacrificed, and the OBs were quickly removed. OB homogenates were prepared and the total protein concentration was estimated using the Bradford protein assay. Ten micrograms of total proteins per lane were loaded onto 10% PAGE gels with loading buffer. The protein samples were run in the Tris-glycine-SDS system at 80 V for approximately 150 minutes and then transferred to polyvinylidene difluoride (PVDF) membranes in Tris-glycine containing 20% methanol at 80 V for 40 minutes. Then, the membranes were blocked by 5% non-fat milk in TBST for one hour and incubated with a primary antibody diluted in 5% non-fat milk or 3% BSA overnight. After being washed in TBST for 3×10 minutes, the membranes were incubated with an HRP-conjugated secondary antibody for 90 minutes at room temperature and were then washed again. The proteins were detected with a chemiluminescent substrate kit (Thermo Scientific, 34080). After stripping the membranes, GAPDH or other antibodies were detected following the same procedure. The Western blot data were imaged and analyzed using the FluorChem HD2 system (NatureGene Corp., USA).

### Odor exposure

Adult C57BL/6 mice were placed in a cage (size: 30 cm×30 cm×30 cm). Isoamyl acetate (1×10^−2^ diluted in mineral oil) or fresh air (for control group) was delivered through a plastic tube at a rate of 0.2 L/min. Another plastic tube was connected to a pump at the opposite position in the cage to draw off the odor so that the odor did not remain in the cage during the exposure intervals. The odor was delivered for 2 minutes with an 8-minute interval between the deliveries. The short-term stimulation lasted for 2 hours, and the long-term stimulation lasted for 8 hours. After the odor exposure procedure, the mice were anesthetized with urethane and the OBs were quickly removed for Western blot analysis.

### In vivo electrophysiology recording

The recordings were conducted on freely breathing mice anesthetized with urethane (1.4 g/kg). The skull was exposed, and a small hole was drilled over the OB for electrode placement. For spontaneous LFP recording, an electrode (PFA-coated, diameter 100 µm) was placed into the granule cell layer. The reference electrode was placed at the skull screw above the cortical hemisphere. For vehicle or TDZD-8 administration, a PE tube was placed into the abdominal cavity before electrophysiology recording. The LFP signals were amplified (×2000, Dagan) and digitized at 2000 Hz (µ-1401, CED). The spontaneous baseline was recorded for 15 minutes, and then either vehicle or TDZD-8 was intraperitoneally delivered through the pre-implanted PE tube. To reduce the possibility that the vehicle/TDZD-8 administration procedure might affect the electrophysiological signals, spontaneous baseline data were recorded half an hour after vehicle/TDZD-8 delivery. After the recording, a current (30 µA) was applied to mark the electrode recording sites. Then the mice were transcardially perfused, and the electrode sites were verified by DAPI staining.

### Analysis of the electrophysiological data

All the 15 minutes’ recording data were used for analysis. Power spectra data were processed in Spike2 (CED) using a fast Fourier transformation of LFPs. The spectrum powers of the four frequency bands, 1–12 Hz (Theta), 12–35 Hz (Beta), 35–60 Hz (low Gamma), 60–90 Hz (high Gamma), were calculated for further analysis. For each mouse, pre-vehicle or pre-TDZD-8 treatment spontaneous oscillatory activities were set at a value of 1, and post-vehicle- or post-TDZD-8-treated data were set relative to the pre-treated data from the same mouse. These data were further analyzed using SPSS 13 by a paired *t*-test (pre- and post-treatment) and independent-Samples *t*-Test (vehicle- and TDZD-8-treatment).

### Cannulas Implants

The mice were anesthetized with intraperitoneal pentobarbital, and stainless steel cannulas (26 gauge, PlasticsOne) were stereotactically implanted bilaterally into both of their olfactory bulbs. After surgery, the mice were allowed to recover for three weeks before undergoing any additional procedures.

### Animal behavior experiments

Six odors were selected for the animal behavior tests, including pentanol, vanillin, geranialdehyde, acetophenone, heptanone and isoamyl acetate. The odors were diluted 1×10^−3^ in mineral oil and applied to cotton applicator sticks. Two hours prior to the animal behavior procedure, 1 µl of TDZD-8 (10 mM dissolved with 50% DMSO in normal saline; Sigma; T8325) or vehicle was bilaterally injected into the OB through the pre-implanted cannula with a 33-gauge Hamilton syringe. In a given block, the odor was delivered using cotton sticks for three successive trials of 1 minute each and separated by 1-minute intervals. The investigation time was defined as the duration time of snout-oriented sniffing within 1 cm of the odor cotton sticks. The time that the mouse spent investigating each odor was measured with a stopwatch.

### Behavior data analysis

The analysis procedures for the behavioral data were similar to those described in previous publications [18]. Briefly, the investigation times and latencies of trial one for the six tested odors were pooled for the vehicle- and TDZD-8-treated groups and further analyzed using repeated measure ANOVA. We used a paired T-test to measure the odor habituation abilities of the vehicle- and TDZD-8-treated groups. Normal habituation ability was defined as a significant difference in the duration times of trial 1 to trial 3 for each odor. In addition, all raw investigation times were divided by the maximum time per animal for each odor. Thus, the maximum investigation duration was assigned as 1, and the lesser investigation times are presented as a fraction [18]. These normalized data were set as the habituation index within each group and analyzed by repeated measure ANOVA. Finally, to determine the effects of GSK3β activity on odor cross-habituation (discrimination ability), the normalized investigatory values from all third-trial odor presentations were subtracted from the following first-trial of new odor presentations. These values were set as the “cross-habituation index” [18], [45] and analyzed by repeated measure ANOVA.

## Supporting Information

Figure S1 **The broad expression of GSK3β in the adult mouse OB.** A: Low magnification view of GSK3β staining signal in the adult OB. White boxes indicate the different layers of OB shown in B–D at higher magnification. B: The glomerular layer (GL). C: The mitral cell layer (MCL). D: The granule cell layer (GCL). Scale bar: A, 200 µm; B–D, 50 µm.
(TIF)

Figure S2 **The** **constitutive phosphorylation of Y216-GSK3β in the adult mouse OB.** A: The majority of mitral cells are positive for Y216-GSK3β (yellow arrow). B: The granule cells (yellow arrow) express Y216-GSK3β. C: Western blot shows that Y216-GSK3β is abundantly detected in the adult mouse OB. Scale bar: 50 µm.
(TIF)

Figure S3 **Expression levels of Syn and GAP43 at different ZnSO_4_ irrigation stages.** Syn and GAP43 expression levels in the OB were all significantly reduced two weeks and one month post ZnSO_4_ irrigation (5–6 mice per group, all P<0.05) and nearly recovered 4 months post-ZnSO_4_ irrigation (5 mice, P>0.05). Syn, Synaptophysin; GAP43, growth associated protein 43; Con, control; 2W, 2 weeks; 1M, 1 month; 4M, 4 month. * P<0.05, ** P<0.01, *** P<0.001. Not significant (N.S.). Data are shown as the mean ± SEM.
(TIF)

Figure S4 **Filtered electrophysiology signals of different frequency bands from the vehicle- and TDZD- treatment mice.** Signals in all bands in the granular cell layer are decreased after TDZD-8 treatment. Theta, 1–12 Hz; Beta, 12–35 Hz; low-Gamma, 35–60 Hz; and high-Gamma, 60–90 Hz.
(TIF)

Figure S5 **Electrophysiological recordings of the spontaneous oscillatory activity two hours post vehicle- and TDZD-8-treatement in the OB.** The relative powers are not significantly different in the pre- and post-vehicle treated mice (A, n = 10, P = 0.245), but significantly different between pre- and post- TDZD-8 mice (B, n = 10, P<0.001). Not significant (n.s.). *** P<0.001. The group data shown are the average ± SEM.
(TIF)
