# Supplementary figures and images for: Roles of GSK3β in Odor Habituation and Spontaneous Neural Activity of the Mouse Olfactory Bulb
Source: PLoS One. 2013 May 3;8(5):e63598. doi: 10.1371/journal.pone.0063598 (PMC3643914; doi:10.1371/journal.pone.0063598)

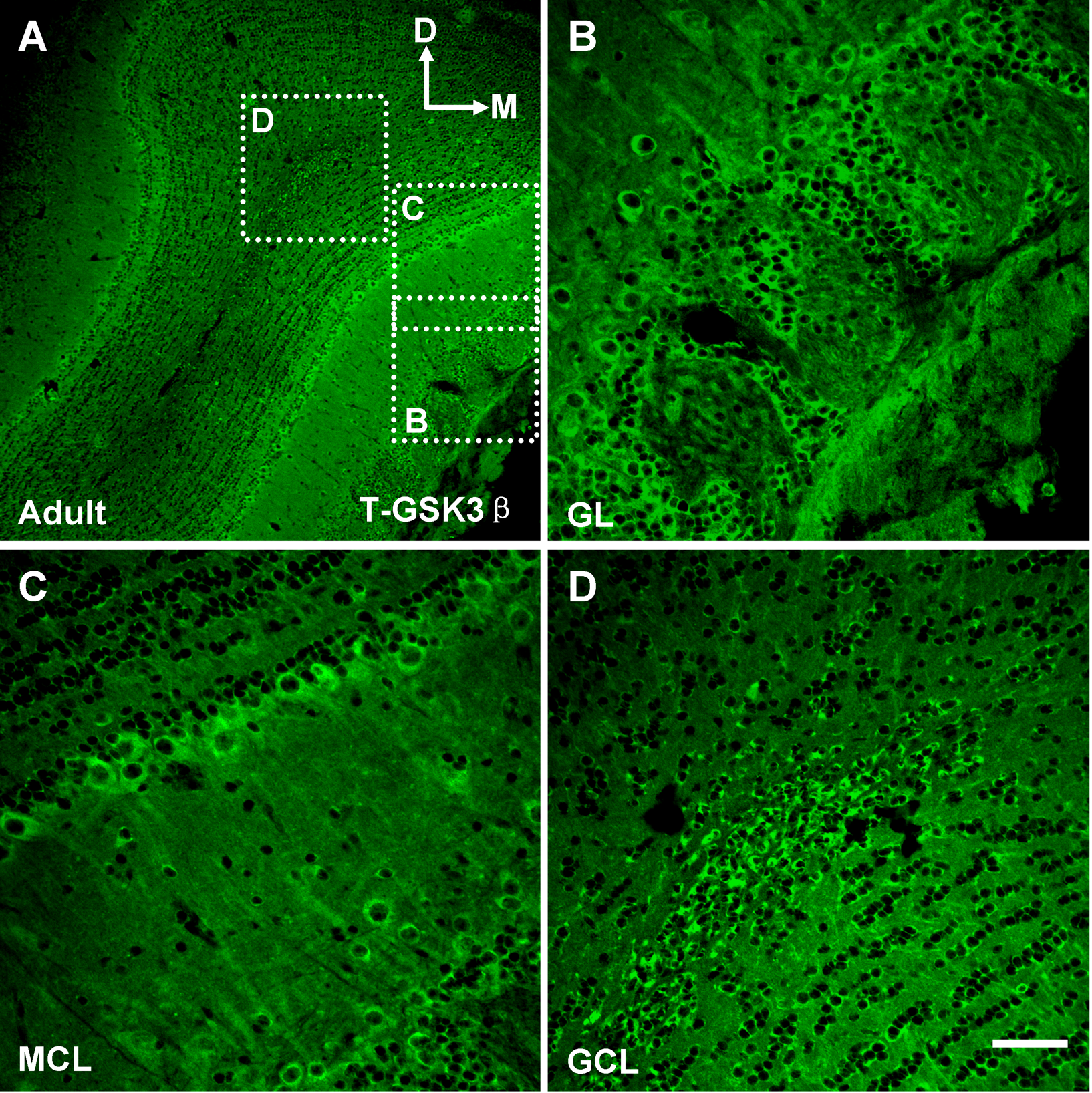

Supplement: Figure S1 — The broad expression of GSK3β in the adult mouse OB. A: Low magnification view of GSK3β staining signal in the adult OB. White boxes indicate the different layers of OB shown in B–D at higher magnification. B: The glomerular layer (GL). C: The mitral cell layer (MCL). D: The granule cell layer (GCL). Scale bar: A, 200 µm; B–D, 50 µm. (TIF) [file pone.0063598.s001.tif]

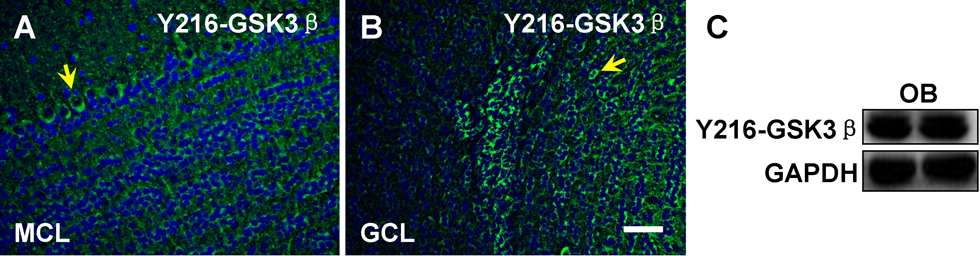

Supplement: Figure S2 — The constitutive phosphorylation of Y216-GSK3β in the adult mouse OB. A: The majority of mitral cells are positive for Y216-GSK3β (yellow arrow). B: The granule cells (yellow arrow) express Y216-GSK3β. C: Western blot shows that Y216-GSK3β is abundantly detected in the adult mouse OB. Scale bar: 50 µm. (TIF) [file pone.0063598.s002.tif]

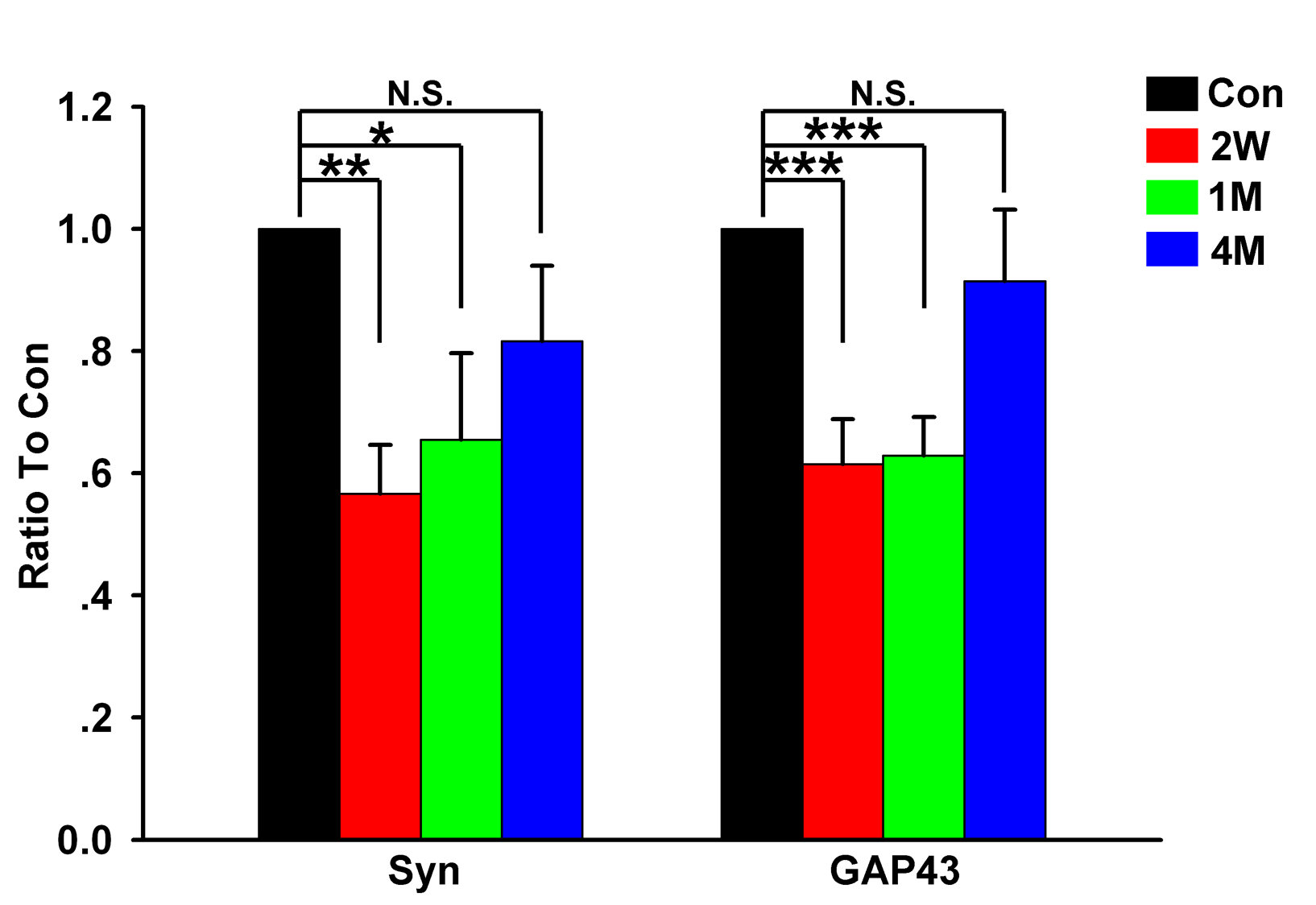

Supplement: Figure S3 — Expression levels of Syn and GAP43 at different ZnSO4 irrigation stages. Syn and GAP43 expression levels in the OB were all significantly reduced two weeks and one month post ZnSO4 irrigation (5–6 mice per group, all P<0.05) and nearly recovered 4 months post-ZnSO4 irrigation (5 mice, P>0.05). Syn, Synaptophysin; GAP43, growth associated protein 43; Con, control; 2W, 2 weeks; 1M, 1 month; 4M, 4 month. * P<0.05, ** P<0.01, *** P<0.001. Not significant (N.S.). Data are shown as the mean ± SEM. (TIF) [file pone.0063598.s003.tif]

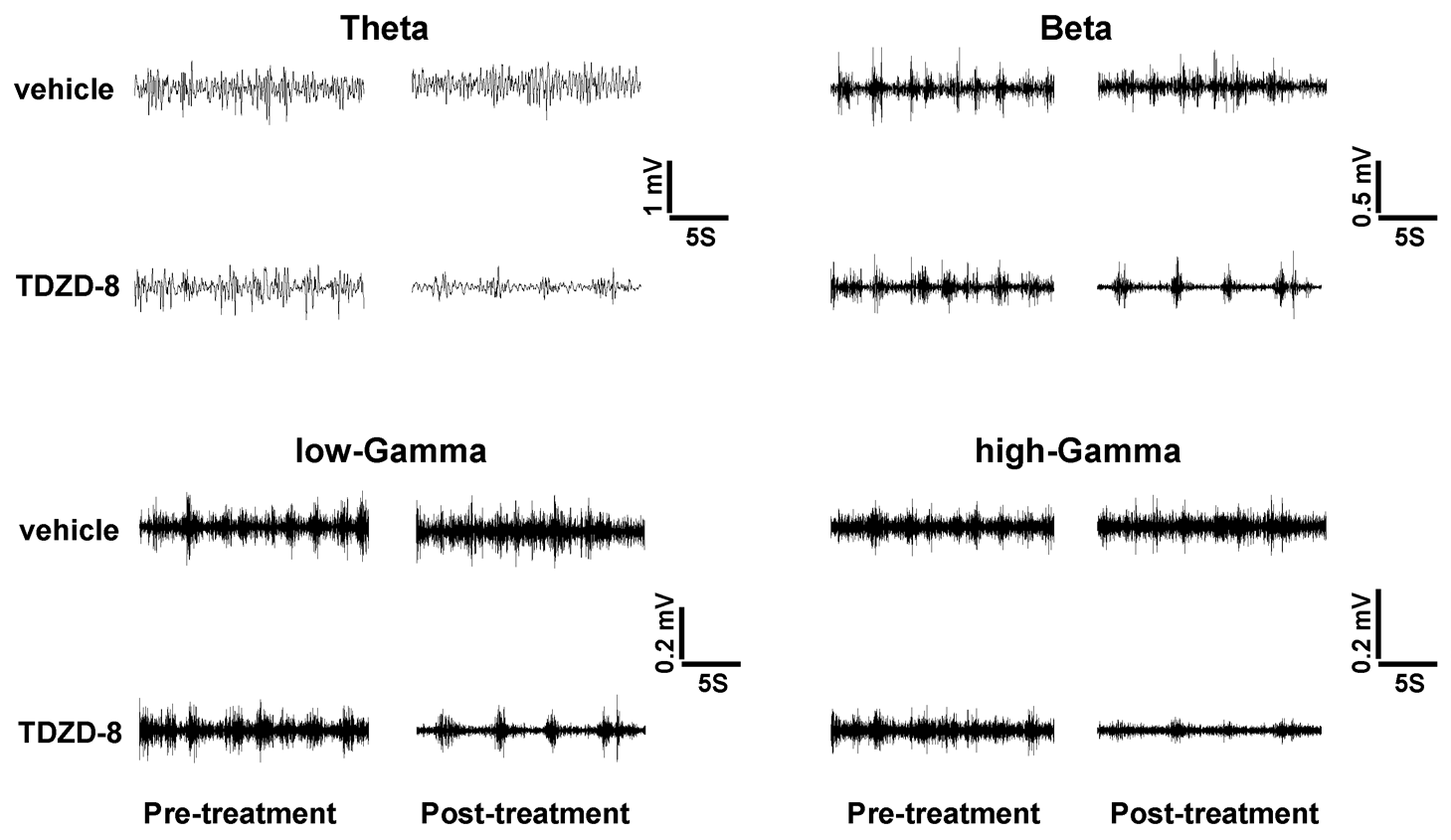

Supplement: Figure S4 — Filtered electrophysiology signals of different frequency bands from the vehicle- and TDZD- treatment mice. Signals in all bands in the granular cell layer are decreased after TDZD-8 treatment. Theta, 1–12 Hz; Beta, 12–35 Hz; low-Gamma, 35–60 Hz; and high-Gamma, 60–90 Hz. (TIF) [file pone.0063598.s004.tif]

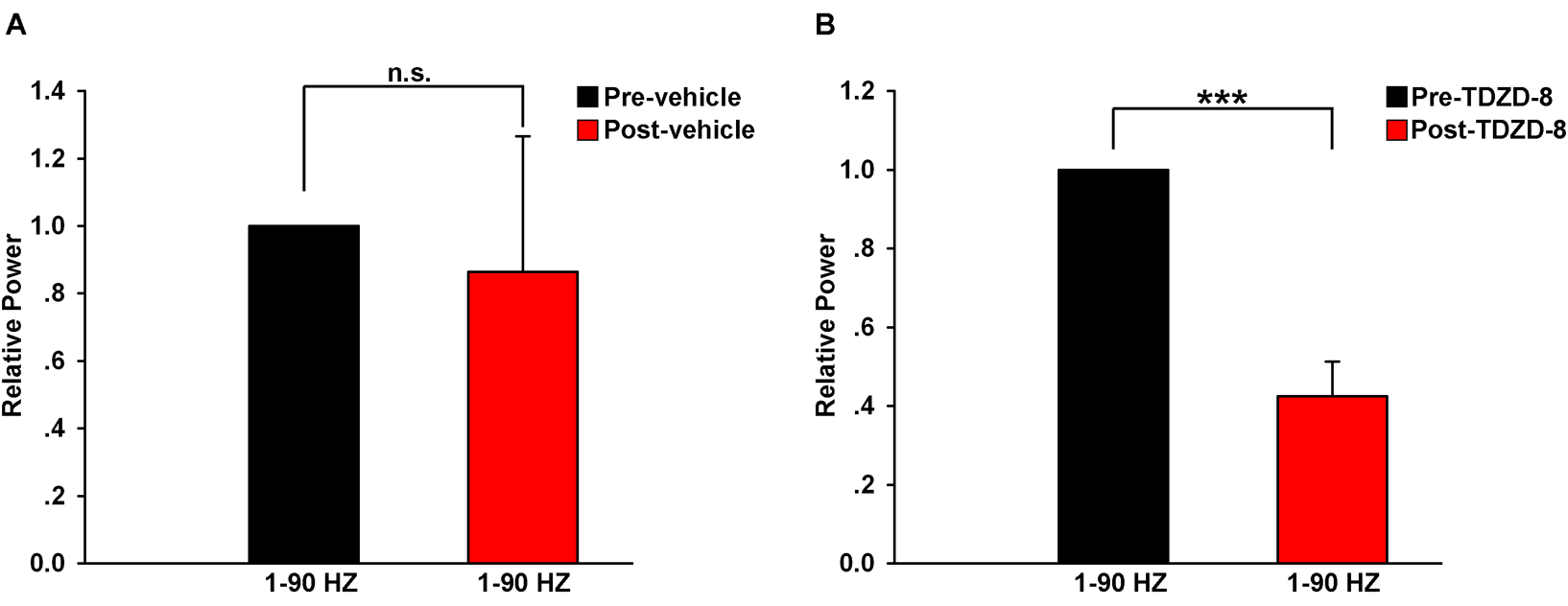

Supplement: Figure S5 — Electrophysiological recordings of the spontaneous oscillatory activity two hours post vehicle- and TDZD-8-treatement in the OB. The relative powers are not significantly different in the pre- and post-vehicle treated mice (A, n = 10, P = 0.245), but significantly different between pre- and post- TDZD-8 mice (B, n = 10, P<0.001). Not significant (n.s.). *** P<0.001. The group data shown are the average ± SEM. (TIF) [file pone.0063598.s005.tif]
